# Distinctive Features of PipX, a Unique Signaling Protein of Cyanobacteria

**DOI:** 10.3390/life10060079

**Published:** 2020-05-28

**Authors:** Jose I. Labella, Raquel Cantos, Paloma Salinas, Javier Espinosa, Asunción Contreras

**Affiliations:** Dpto. Fisiología, Genética y Microbiología, Universidad de Alicante, 03690 Alicante, Spain; ls.joseignacio@ua.es (J.I.L.); raquel.cantos@ua.es (R.C.); paloma.salinas@ua.es (P.S.); javier.espinosa@ua.es (J.E.)

**Keywords:** cyanobacteria, signal transduction, nitrogen regulation, interaction network, synteny network

## Abstract

PipX is a unique cyanobacterial protein identified by its ability to bind to PII and NtcA, two key regulators involved in the integration of signals of the nitrogen/carbon and energy status, with a tremendous impact on nitrogen assimilation and gene expression in cyanobacteria. PipX provides a mechanistic link between PII, the most widely distributed signaling protein, and NtcA, a global transcriptional regulator of cyanobacteria. PII, required for cell survival unless PipX is inactivated or down-regulated, functions by protein–protein interactions with transcriptional regulators, transporters, and enzymes. In addition, PipX appears to be involved in a wider signaling network, supported by the following observations: (i) PII–PipX complexes interact with PlmA, an as yet poorly characterized transcriptional regulator also restricted to cyanobacteria; (ii) the *pipX* gene is functionally connected with *pipY*, a gene encoding a universally conserved pyridoxal phosphate binding protein (PLPBP) involved in vitamin B6 and amino acid homeostasis, whose loss-of-function mutations cause B6-dependent epilepsy in humans, and (iii) *pipX* is part of a relatively robust, six-node synteny network that includes *pipY* and four additional genes that might also be functionally connected with *pipX*. In this overview, we propose that the study of the protein–protein interaction and synteny networks involving PipX would contribute to understanding the peculiarities and idiosyncrasy of signaling pathways that are conserved in cyanobacteria.

## 1. Introduction

Cyanobacteria, phototrophic organisms that perform oxygenic photosynthesis, constitute an ecologically important phylum that is responsible for the evolution of the oxygenic atmosphere, and are the main contributors to marine primary production [1]. Their photosynthetic lifestyle and ease of cultivation make them ideal production systems for a number of high-value compounds, including biofuels [2]. However, cyanobacteria have developed sophisticated systems to maintain the homeostasis of carbon/nitrogen (reviewed by [3,4]), the two most abundant nutrient elements in all living forms; therefore, understanding the regulatory mechanisms affecting their metabolic balance is of paramount importance from the biotechnological as well as the environmental points of view. 

Cyanobacteria can use different nitrogen sources that are then converted into ammonium and incorporated via the glutamine synthetase-glutamate synthase (GS-GOGAT) cycle into carbon skeleton 2-oxoglutarate (2-OG) for the biosynthesis of amino acids and other N-containing compounds. The 2-OG, a universal indicator of the intracellular carbon-to-nitrogen balance [5,6], appears to be particularly suitable for this role in cyanobacteria, because the lack of 2-OG dehydrogenase results in the accumulation of 2-OG during nitrogen starvation [7]. Recently, a role as an antioxidant agent involved in reactive oxygen species (ROS) homeostasis has also been proposed for 2-OG in cyanobacteria [8]. 

In bacteria and plants, 2-OG is sensed by the widely distributed signal transduction protein PII, which is encoded by *glnB* and is a homotrimer with one binding site per subunit for 2-OG. PII regulates the activity of proteins implicated in nitrogen metabolism by direct protein–protein interactions [3]. The first two PII receptors in cyanobacteria [9,10] were identified using the yeast two-hybrid system [11] to search for proteins interacting with PII in *Synechococcus elongatus* PCC7942 (hereafter *S. elongatus*). One of the identified proteins was the enzyme N-acetyl-glutamate-kinase (NAGK), which catalyzes a key regulatory step in the biosynthesis of arginine that is stimulated by PII in cyanobacteria and plant chloroplasts [10,12,13]. The other one was a small and previously unknown protein of 89 amino acids that was named PipX (PII interacting protein X). PipX was also found as prey in yeast two-hybrid searches with NtcA, the global transcriptional regulator involved in nitrogen assimilation in cyanobacteria [14,15]. Since PII and NtcA are 2-OG receptors, PipX appeared to be a novel component of the nitrogen signal transduction pathway in this phylum. Subsequent work confirmed the role of PipX as a regulatory link between PII and NtcA, and unraveled the functional and structural details of the PipX–PII and PipX–NtcA interactions [16,17,18,19,20,21]. Although most of the significant advances relate to *S. elongatus*, experimental and *in silico* evidence supports conservation of the same nitrogen-related regulatory interactions of PipX in the cyanobacterial phylum [21,22,23,24,25,26,27,28].

Additional “guilty by association” approaches have extended the physical and functional networks of PipX. Yeast three-hybrid searches with PipX–PII as bait resulted in the identification of the cyanobacterial transcriptional regulator PlmA as an interacting protein [29], while co-expression and synteny approaches functionally connected PipX with PipY, a conserved pyridoxal phosphate-binding protein involved in amino/keto acid and pyridoxal phosphate (PLP) homeostasis [30,31,32].

## 2. The Complex Nitrogen Signaling Network of Cyanobacteria

### 2.1. PII and PipX as Dynamic Hubs of an Extended Protein Interaction Network 

**Figure 1:**
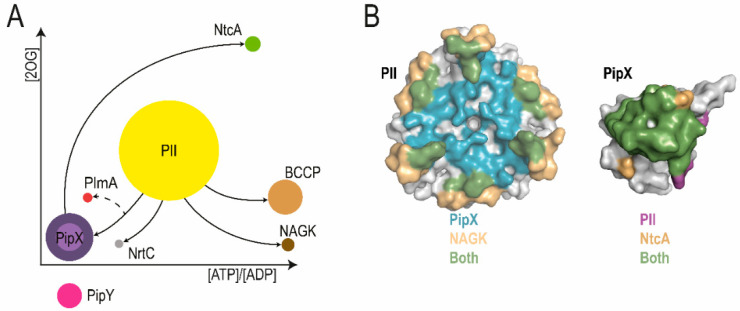
The PipX interaction network. (**A**) PII and PII PipX interactions, according to the concentration spectra of 2-OG and ATP/ADP ratios. Molecular players are illustrated as circles whose sizes, drawn to scale, refer to the number of protein molecules, according to [34], taking into account their quaternary structure: 20.078 PII trimers, 4.560 PipX monomers, and 1.520 PipX trimers (dark- and light-colored circles, respectively), as well as 510 NtcA dimers, 200 PlmA dimers, 160 NrtC monomers, 359 NAGK hexamers, and 2409 biotin carboxyl carrier protein (BCCP) monomers. The arrows go from hub to target proteins, with PipX being considered as a PII target. The position of the arrowheads (towards target proteins) indicates the conditions favoring the corresponding complexes, one of which is a ternary complex (PII–PipX–PlmA). A dashed arrow is used for PlmA interaction with PipX–PII, which has not been characterized at the molecular level but should be favored by relatively low 2-OG levels and low ATP/ADP ratios. PipY (1.275 monomers), for which no protein–protein interactions are known, is shown outside the graphical representation. (**B**) The three-dimensional (3D) structures of PII (left) and PipX (right), with surfaces colored according to the area of interaction, with NAGK (yellow) and PipX (blue) for PII, and with PII (purple) and NtcA (yellow) for PipX. (Adapted from [35] (**A**) and [16] (**B**)).

PII and PipX mediate protein–protein interactions with regulatory targets that include transcriptional regulators, enzymes, and transporters involved in nitrogen and carbon assimilation, forming an ever-growing and dynamic interaction network that largely depends on PII effectors. Thus, the levels of 2-OG and the ATP/ADP ratio are the main intracellular signal molecules determining protein–protein interactions [33]. In addition, this complex network is necessarily affected by the relative abundance of the different components. In *S. elongatus*, estimations of the numbers of chains of the relevant proteins can be obtained from massive proteomic studies [34]. In a comprehensive scheme, Figure 1A illustrates the nitrogen interaction network and the position of PII and PipX as regulatory hubs, integrating information on the relative abundance of the protein components and the levels of signal molecules determining protein–protein interactions. For the sake of simplicity, only protein components whose interactions with the hubs have been characterized to a certain extent (discussed in this section) are included in the illustration. The relative abundance of PipY, considered a member of the corresponding PipX–PII regulatory network (see below), is also illustrated for comparison. 

PII perceives metabolic information by the competitive binding of ATP or ADP and by the synergistic binding of ATP and 2-OG [36]. The PII trimer has three binding sites for ATP/ADP (in some species AMP) and 2-OG. PII binds to NAGK, stimulating its activity and promoting nitrogen storage as arginine in cyanobacteria and plants [10,37,38,39]. When abundant, 2-OG binds to MgATP-complexed PII, triggering conformational changes that prevent the interaction of PII with either NAGK or PipX [9,37]. In the absence of 2-OG, only the ATP/ADP ratio and concentration of ADP directs the competitive interaction of PII with these targets in vitro. PipX increases the affinity of PII for ADP, and, conversely, the interaction between PII and PipX is highly sensitive to fluctuations in the ATP/ADP ratio [40]. Since the same surface of PII binds either NAGK or PipX (Figure 1B), these proteins do not form ternary complexes. Although competition between NAGK and PipX for PII binding can be observed in vitro [40,41], the great excess of PII over these two binding partners and the presence of additional actors makes competition a less likely scenario in vivo. 

PII also binds to the biotin carboxyl carrier protein (BCCP) of acetyl-CoA carboxylase (ACCase), inhibiting its ability to control acetyl-CoA levels in organisms in which PII is present [42]. PII-dependent inhibition of nitrate transport, known to occur after the addition of ammonium to nitrate-containing cultures or the transfer of cultures to darkness, requires interaction of PII with the NrtD and NrtC subunits of the nitrate transporter (NRT) [28,43,44,45]. Other recently discovered PII receptors in *Synechocystis* sp. PCC 6803 are the ammonium (Amt1) and urea (UrtE subunit involved) transporters and two proteins of as yet unknown functions (Sll0944/DUF1830/_0891 and Ssr0692) [43].

2-OG stimulates complex formation between the global transcriptional regulator NtcA, a CRP-like protein, and PipX [9], as well as the binding of NtcA to target sites [46] and transcription activation in vitro [47]. The PipX–NtcA complex consists of one active (2-OG bound) NtcA dimer and two PipX molecules. Each NtcA subunit binds one PipX molecule in such a way that it stabilizes the active NtcA conformation and probably helps to recruit RNA polymerase without providing extra DNA contacts [16,48]. Comparative studies of cyanobacterial NtcA and CRP proteins and their interactions with DNA and effectors (2-OG and cAMP) provided additional details of NtcA–PipX functional interactions [22]. 

Importantly, PipX provides a mechanistic link between PII signaling and gene expression, depending on NtcA, which controls a large regulon in response to nitrogen limitation [23,49]. PipX uses the same surface from its N-terminal domain to bind to either 2-OG-bound NtcA (Figure 1B), stimulating DNA binding and transcriptional activity, or to 2-OG-free PII, to form PipX–PII complexes [16,21]. Here the relative abundance of the excess PII over PipX provides a predictable scenario of competition with NtcA for PipX in vivo, at least under physiological conditions in which the affinity of PipX for NtcA is not optimal. 

PII-PipX complexes interact with the transcriptional factor PlmA [29], suggesting a role of nitrogen regulators in the transcriptional control of the yet unknown PlmA regulon. The main contacts of PlmA with PII-PipX complexes appear restricted to surface-exposed elements of PipX, specifically one residue in its Tudor Like Domain (TLD/KOW) and the C-terminal helices, which acquire an open conformation when bound to PII [50]. Therefore, the PipX determinants for binding to PlmA appear be very different from those involved in PipX–NtcA complexes.

**Figure 2:**
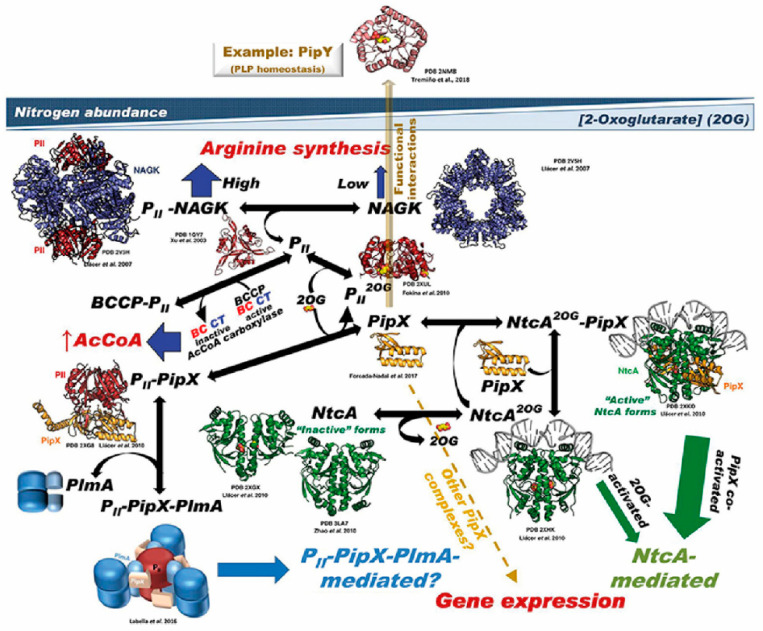
Summary of the PII–PipX–NtcA network of *S. elongatus*. The network illustrates its different elements and complexes depending on nitrogen abundance (inversely related to 2-OG level), as well as the structures of the macromolecules and complexes formed (when known). For PlmA (dimer in darker and lighter blue hues for its dimerization and DNA-binding domains, respectively) and its complex, the architectural coarse model proposed [29] is shown, with the C-terminal helices of PipX (schematized in the extended conformation) illustrated as pink-colored and the two PII molecules in dark red. The DNA complexed with NtcA and with NtcA-PipX is modeled from the structure of DNA–CRP [16], since no DNA–NtcA structure has been reported. BCCP is the biotin carboxyl carrier protein of bacterial acetyl CoA carboxylase (abbreviated AcCoA carboxylase); the other two components of this enzyme, biotin carboxylase and carboxyl transferase, are abbreviated BC and CT, respectively. No structural model of BCC has been shown, because the structure of this component has not been determined in *S. elongatus*, and also because the structures of this protein from other bacteria lack a disordered, 77-residue N-terminal portion that could be highly relevant for interaction with PII. The broken yellow arrow highlights the possibility of further PipX interactions not mediated by NtcA or PII–PlmA, resulting in changes in gene expression [49]. The solid, semi-transparent, yellowish arrow emerging perpendicularly from the flat network symbolizes the possibility of functional interactions of PipX not mediated by physical contacts between the macromolecules involved in the interaction, giving as an example the functional interaction with PipY. Its position outside the network tries to express this different type of interaction (relative to the physical contacts shown in the remainder of the network), as well as to place it outside the field of 2-OG concentrations (taken from [33]).

A comprehensive summary of relevant complexes and interactions involving PipX and PII proteins are illustrated in Figure 2. The reader is referred to [33] for additional structural information and details on complex formation that are omitted in this review. 

### 2.2. Role of PII and PipX in Cyanobacterial Survival 

While genetic inactivation of *pipX* appears to have little impact in *S. elongatus* survival under standard laboratory conditions [17,51], genetic inactivation of *glnB* is not viable in a wild-type background. Unsuccessful attempts to completely segregate *glnB* null alleles thus indicate that the PII protein is essential for survival, a finding in close agreement with the importance of *glnB* genes in many other microorganisms, where inactivation leads to severe growth defects or lethality. Interestingly, the metabolic basis of *glnB* deficiency in the studied microorganisms seems to be diverse (discussed in [18]). In cyanobacteria, the essentiality of PII appears to be related with the importance of PipX–PII complex formation, presumably to counteract PipX functions. In line with this, a small reduction in PipX levels suppresses the need for PII for the survival of *S. elongatus* [18,19,20]. The importance of PII for cell survival is even more pronounced in nitrogen-rich media [51], conditions in which the inhibitory effect of 2-OG on PipX–PII interactions would not take place.

It is worth noting that cyanobacterial genomes always contain at least as many copies of *glnB* as of *pipX*, with duplications of *pipX* correlating with duplications of *glnB* [52], in line with the idea that a relatively high ratio of PII over PipX is required to counteract unwanted interactions with low-affinity PipX partners. In *Synechococcus* WH5701, a cyanobacterium with two PipX and two PII-like proteins, differences in affinities between PII and PipX paralogs and their binding partners—PipX-I, PipX-II, GlnB-A, GlnB-B, NAGK, or NtcA—presumably increases their regulatory potential. Therefore, by integrating multiple signaling pathways, PII and PipX are likely to play currently unknown roles in adaptation-to-environment situations faced by cyanobacteria.

The basis of the phenomenon that we have called “PipX toxicity” in the absence of PII has been explored by mutational analyses, which initially took advantage of point mutations, either identified as spontaneous suppressors in *glnB* strains or generated in the course of our investigations on PipX determinants for interactions with PII or NtcA. The question of whether PipX toxicity is due to over-activation of the NtcA regulon when there is not enough PII to prevent PipX binding to NtcA has been explored by subjecting *pipX* mutant derivatives to co-activation assays (reporting from NtcA activated promoters *glnB* and *glnN*) and to transcriptomic analysis (in the case of two gain-of-functions mutations in the contexts of NtcA coactivation and PipX toxicity) [19,20,49]. However, a cause–effect relationship between PipX toxicity and the over-expression of NtcA gene targets could not be established [20], and it is thus possible that both over-expression of NtcA gene targets and interactions of PipX with a third partner may contribute to PipX toxicity. In the later context, genetic and transcriptomic analyses have also suggested additional regulatory targets of PipX [49]. 

**Figure 3:**
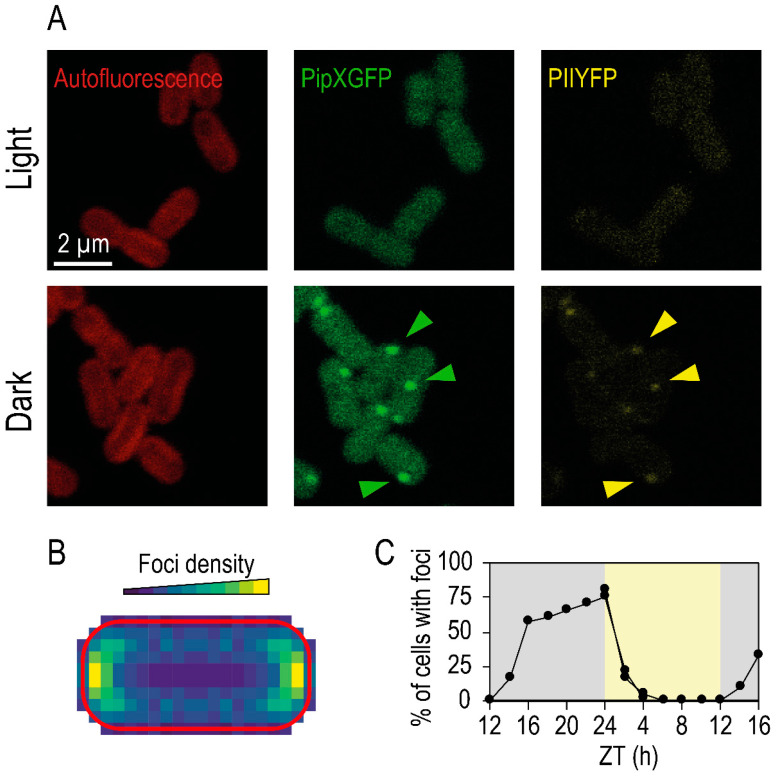
PipXGFP and PIIYFP fusion proteins localize into foci in darkness. (**A**) *S. elongatus* cells were imaged from cultures grown with nitrate in the light or dark (8 h). The autofluorescence (red), PipXGFP (green), and PIIYFP (yellow) signals are shown as indicated. (**B**) Heatmap of PipXGFP distribution relative to the cell autofluorescence border (in red). Colors represent the density of foci at each cellular position. (**C**) Percentage of the cell population that showed PipXGFP foci in diurnal conditions (light and dark conditions corresponding to yellow and gray regions, respectively). Cells were entrained in light–dark cycles and sampled every 2 hours in either the light (ZT 0–12) or dark (ZT 12–24). ZT (Zeitgeber time) refers to the time relative to “lights on” (adapted from [35]).

Interestingly, PII and PipX display distinct localization patterns during diurnal cycles, co-localizing into foci located at the periphery and cell poles during dark periods (Figure 3), a process dependent on energy levels [35]. These dynamic regulatory interactions would play circadian-independent roles in the attenuation of transcriptional activity and other functions during dark periods, while facilitating the return to the essential, and energy costly, light-dependent or light-induced activities. 

### 2.3. PipX Role in Gene Expression and Interactions with the Unique Transcriptional Regulators NtcA and PlmA

In cyanobacteria, multiple metabolic and developmental processes are induced by nitrogen starvation. NtcA, the global regulator for nitrogen control, activates genes involved in nitrogen assimilation, heterocyst differentiation, and acclimation to nitrogen starvation [14,15,53,54,55,56]. The interaction between PipX and NtcA is known to be relevant under nitrogen limitation for the activation of NtcA-dependent genes in *S. elongatus* and *Anabaena* sp. Strain PCC 7120 [9,17,24,26], and presumably in *Prochlorococcus* [27].

From a combination of genetic, transcriptomic, and multivariate analyses, we previously obtained groups of genes differentially regulated by PipX that have improved the definition of the consensus NtcA binding motif for *S. elongatus* and provided further insights into the function of NtcA–PipX complexes. Importantly, additional groups of genes that are differentially regulated by PipX suggested the involvement of PipX in NtcA-independent regulatory pathways, indicating that PipX is involved in a much wider interaction network affecting nitrogen assimilation, translation, and photosynthesis [49].

PlmA, the other transcriptional regulator interacting with PipX (as part of PipX–PII complexes) belongs to the GntR super-family of transcriptional regulators [57]. It is involved in photosystem stoichiometry in *Synechocystis* sp. PCC 6803 [58], plasmid maintenance in *Anabaena* sp. strain PCC 7120 [59], and in regulation of the highly conserved cyanobacterial sRNA YFR2 in marine picocyanobacteria [60]. Recently, it has been shown that in *Synechocystis* sp. PCC 6803, PlmA is reduced by the thioredoxin TrxM [61], suggesting that its function may depend on the redox status. 

**Figure 4:**
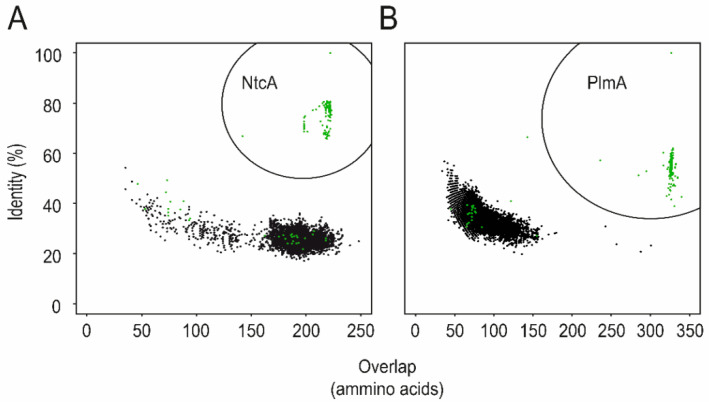
The NtcA (**A**) and PlmA (**B**) subfamilies. Bidirectional blast queries with *S. elongatus* PlmA and NtcA sequences as queries resulted in 33.903 and 102.738 sequences homologous to NtcA and PlmA, respectively, from the Refseq genomic bacterial database. Horizontal and vertical axes represent, respectively, the amino acid overlap and identity of each hit. Green dots, accounting for 285 (in **A**) and 264 (in **B**) hits, were retrieved from cyanobacterial genomes. Those dots corresponding to cyanobacterial NtcA and PlmA orthologs (238 and 234 hits, respectively) are encircled (adapted from [29]).

NtcA and PlmA belong to large families of transcriptional regulators (CRP and GntR). Both are universally present in cyanobacteria, and their presence is also restricted to this group (Figure 4), as is the case with PipX. Therefore, the three proteins are hallmarks of cyanobacteria [29,57,59]. In contrast to the already abundant structural and functional details about NtcA, very little is still known about PlmA, but its unique and exclusive distribution within cyanobacteria suggest that PlmA functions are important to cope with regulatory signals and metabolites that are relevant in the context of photosynthesis.

## 3. Regulatory Connections between PipX and the Conserved PLP-Binding Protein PipY

### 3.1. The Tight Link between pipX and pipY Genes in Cyanobacteria

**Figure 5:**
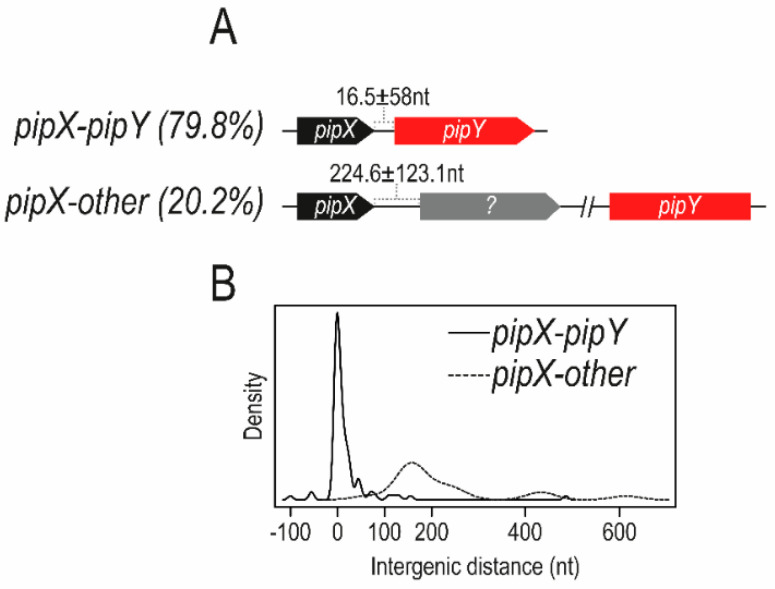
*pipX–pipY* and other gene arrangements. The two possible situations concerning *pipX* and *pipX* genes found in cyanobacterial genomes are represented: *pipX* (black) is followed by *pipY* (red), always in the same orientation (as illustrated by the direction of the arrows), or by apparently non-related and diverse genes (illustrated by a grey arrow). For each situation, the corresponding percentage found in cyanobacterial genomes is shown in brackets. The intergenic distance between *pipX* and downstream genes in cyanobacterial genomes is shown as the mean and standard deviation (A) and density curve (B). (Adapted from [31]).

Inspection of cyanobacterial genomes [30,32] revealed a tight connection between *pipX* and its downstream gene, named *pipY*, encoding a member of the widely distributed family of pyridoxal phosphate (PLP) binding proteins (PLPBP; COG0325), which control vitamin B6 and amino acid cell homeostasis. In *S. elongatus*, where the two genes form an operon, PipX increases expression of either *pipY* or a reporter gene occupying the *pipY* locus [31], thus suggesting the importance of the PipX–PipY balance. On the other hand, and given the relatively low number of genes that form part of polycistronic units (55% for *S. elongatus*), it is significant that, in almost 80% of the cyanobacterial genomes analyzed, *pipX* genes were found adjacent to *pipY* genes (always upstream and in the same orientation), presumably as part of bicistronic *pipX*Y operons. In addition, tight co-regulation and even translational coupling can be inferred by the relatively short or non-existent intergenic distances found between contiguous *pipX* and *pipY* coding sequences, strongly suggesting a functional interaction between PipX and PipY in most, if not all, cyanobacteria. When not adjacent, as in *Synechocystis* strains, *pipX* sequences constitute monocistronic operons. Figure 5 summarizes *in silico* evidence for the tight link between *pipX* and *pipY* genes found in cyanobacteria genomes.

An open question is whether PipX and PipY proteins physically interact in cyanobacteria, which is so far unsupported by yeast two-hybrid assays [30]. However, a putative interaction between PipX and PipY may require another factor(s) present in *S. elongatus* but not in yeast nuclei, and thus PipX–PipY complex formation should not be ruled out yet. 

### 3.2. Common Structural and Functional Features between PipY and Other PLPBP Members 

**Figure 6:**
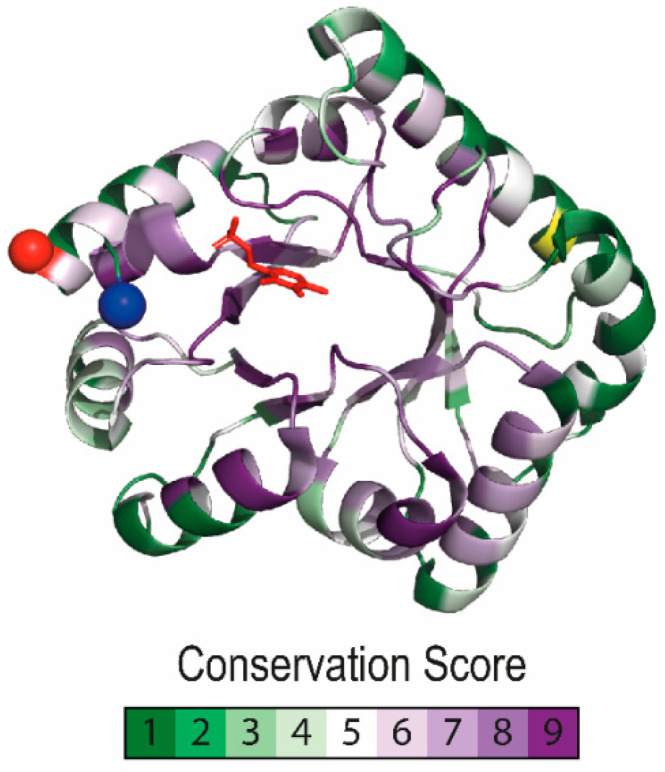
Pyridoxal phosphate binding protein (PLPBP) residue conservation and distinctive structural features. Conservation scores were calculated using CONSURF [65] with COG0325 alignment and a phylogenetic tree from the EGGNOG database as query. The color code illustrates lowest to highest conservation in a green to purple gradient, with yellow for non-informative residues, plotted over the Synpcc7942_2060 (PDB 5NM8) structure. The N-terminal and C-terminal ends are indicated with a red and blue sphere, respectively. The PLP molecule is colored in red.

Structures of PipY homologs from phylogenetically distant organisms, such as yeast (Ybl036c) and cyanobacteria (PipY), have already been experimentally solved or modelled (human PLPHP), and a remarkable conservation is found when both protein sequence and three-dimensional (3D) structures are compared [62,63,64]. PLPBP structures reveal a single domain monomer, folded as the TIM barrel of type III-fold PLP enzymes, with the PLP cofactor solvent exposed. These structures all have an α-helical extension of the C-terminal β-strand binding the phosphate of PLP, which could act as a trigger for PLP exchange (Figure 6) [62,64].

Although our initial interest in PipY stems from its predicted involvement in the cyanobacterial nitrogen regulatory network, the idea that PLPBP members may perform the same basic functions in the context of amino acid and PLP homeostasis in all types of cells has gained strength during the course of our studies. It was concluded that PipY performs the same basic functions inferred for YggS (the *E. coli* homolog), and PLPHP and is therefore a bona fide member of the PLPBP family. In particular, *S. elongatus pipY* mutants [30], like *E. coli yggS* mutants, showed sensitivity to pyridoxine (PN) and an imbalance of the amino/keto acid pools. *pipY* mutants also showed sensitivity to antibiotics targeting essential PLP holoenzymes. Importantly, distantly-related PLPBP members were able to rescue species (or even strain)-specific defects, such as Val overproduction and the PN sensitivity phenotypes of *E. coli yggS* mutants. These two *E. coli yggS* mutant phenotypes were respectively rescued by heterologous expression of orthologs from bacilli (YlmE), yeast (Ybl036c), and humans [66]; and of orthologs from plants (*Zea mays*, *Arabidopsis thaliana*), yeast, and humans [67]. In humans, mutations affecting protein levels or PLP-binding at the PLPHP causes vitamin B6-dependent epilepsy [64,67,68].

Synthetic lethality between PLPBP and PLP holoenzymes, previously reported for *E. coli yggS* and either *glyA* [69,70] or *serA* [71], has also been found between *pipY* and *cysK*, the two most abundant PLP-binding proteins in *S. elongatus* [30]. Synthetic lethality probably reflects the common involvement of the corresponding protein pairs in amino acid and PLP homeostasis, but may also indicate that any relatively abundant PLP-binding protein could fulfill a role as a PLP reservoir, thus helping to cope with PLP toxicity. In this context, it is worth emphasizing that the cofactor in PLPBP is solvent-exposed, and thus is best suited for a role as a PLP delivering modules for essential apo-enzymes in cells.

While the mechanistic details affecting PLPBP-mediated PLP homeostasis and those concerning the regulatory connections between PipY and PipX remain to be elucidated, the apparent recruitment of PipY into the 2-OG-dependent nitrogen interaction network, and its connection with PipX in cyanobacteria (see below), provides a unique opportunity to investigate the functions of a bona fide PLPBP member in the context of a relatively characterized signaling network in a bacterial model system.

### 3.3. The Intriguing Connection between PipY and the Cyanobacterial Nitrogen Network

Two experimental lines of evidence place PipX and PipY within the same regulatory pathway: the two proteins contribute to *S. elongatus* resistance to PLP-targeting antibiotics D-cycloserine and β-chloro-D-alanine, and affect expression of a common set of transcripts. 

In particular, transcriptomic analysis with single and double *pipX* and *pipY* null mutants has revealed the implication of both PipX and PipY in the control of NtcA activated transcripts, where PipY plays a positive role [30]. In the context of NtcA-independent transcripts, PipX and PipY could have similar or opposite effects, suggesting a rather complex regulation [30]. 

In addition, transcriptomic analysis with *pipX* null and gain-of-function mutant derivatives was consistent with PipX having a role as a repressor of many photosynthesis- and translation-related genes independent of NtcA [49]. The model emerging from those analyses was that of PipX implicated in the fine-tuning of different regulons, of which only the NtcA one was anticipated. 

**Figure 7:**
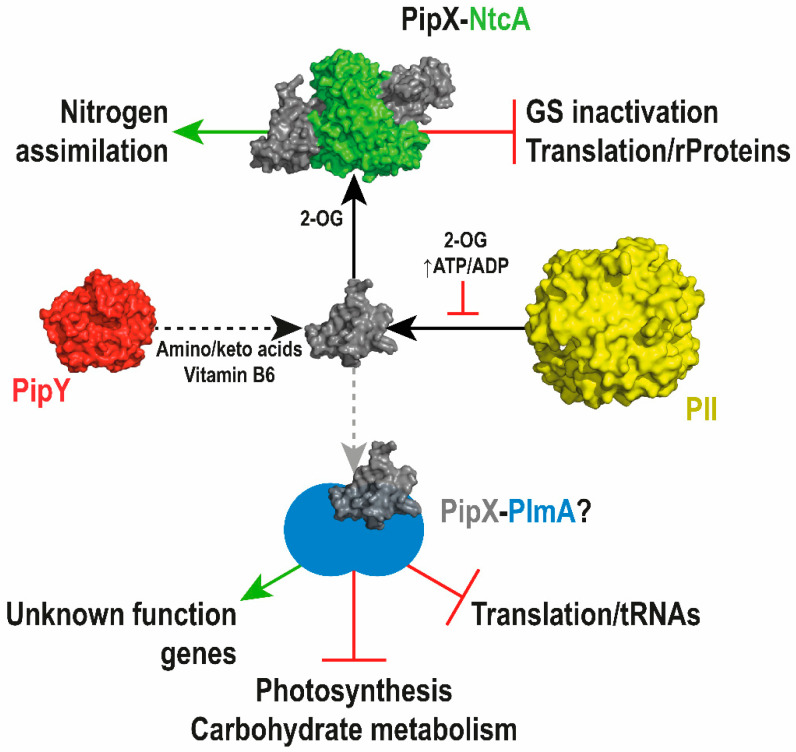
Model of PipX function in transcriptional regulation. PipX, as the NtcA co-regulator, is involved in the (2-OG dependent) activation of multiple operons for nitrogen assimilation, as well as in the repression of translation-related genes and inhibitors of key nitrogen assimilation genes (glutamine synthetase). PipX also works independently of NtcA in the regulation of key processes, including translation, photosynthesis, and carbohydrate metabolism, presumably in a complex with at least one other transcriptional regulator, likely PlmA, whose interaction with PipX depends on PII, and would be regulated by 2-OG and ATP/ADP. PipY may affect all PipX regulatory interactions by altering the levels of amino/keto acids and vitamin B6. Positive and negative regulation are depicted by green arrows and red lines, respectively. Black solid arrows illustrate the formation of complexes driven by the indicated regulatory signals. Dotted arrows indicate putative regulatory interactions by PipY or by hypothetical complex(es) (depicted in a lighter color arrow) that may involve PlmA.

To explain the positive role of PipY on the levels of NtcA-activated transcripts, it is tempting to propose that PipY increases the levels of 2-OG in *S. elongatus* cells—that is, PipY activity would alter nitrogen signaling in cyanobacteria by affecting the levels of 2-OG, a possibility worth considering given the importance of PLPBP proteins in maintaining the balance of the amino/keto acid pools. Similarly, the expression of NtcA-independent transcripts co-regulated by PipX–PipY may also respond to amino/keto acids, or to PLP-related compounds, via a yet-unidentified factor. Here it is tempting to speculate that the transcriptional regulator PlmA may be involved. Figure 7 illustrates our current working model, integrating PipY and PlmA into the PipX regulatory network.

## 4. Unknown Functions of PipX and the Synteny Approach

### 4.1. PipX and NusG Family Proteins Share a Domain Involved in Operon Polarity

**Figure 8:**
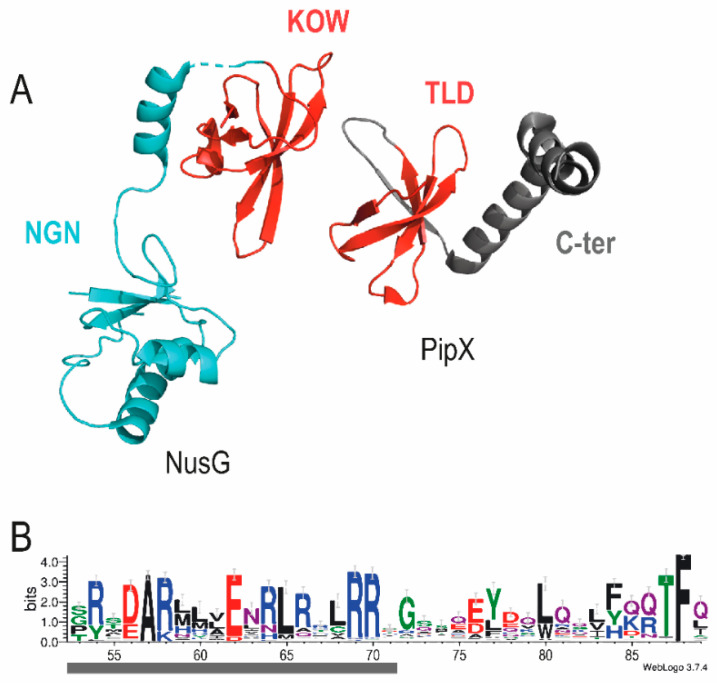
Structural features of PipX, discussed in the context of a possible role in translation regulation. (**A**) Whole length protein structures of NusG from *E. coli* (PDB 5TBZ:J) and PipX from *S. elongatus* (PDB 2XG8:D). TLD/KOW domains are shown in red, the N-terminal domain of NusG (NGN) in blue, and the C-terminal domain of PipX in grey. (**B**) WebLogo of the C-terminal domain of PipX. The first alpha helix containing an arginine-rich patch is underlined in grey (adapted from [31]).

While investigating regulatory connections between *pipX* and *pipY* genes, we became aware of a rather intriguing function of PipX: polarity suppression or the enhancement of *pipY* expression, specifically in *cis* [31]. Most intriguing was the finding that the TLD/KOW structural domain of PipX is shared by NusG proteins, since NusG paralogs typified by RfaH are non-essential, operon-specific bacterial factors involved in the upregulation of horizontally-acquired genes, normally located downstream within the same operon [72,73,74]. Not underestimating the fact that the NusG paralogs act in *trans*, PipX plays the same role over the downstream gene *pipY*, opening the question of whether PipX can also use the TLD/KOW domain to interact with the translation machinery (Figure 8A), as NusG proteins do. On the one hand, key NusG residues contacting ribosomal protein S10 are conserved between PipX and RfaH [31], suggesting the possibility of a similar interaction between PipX and S10. On the other hand, the C-terminal domain of PipX contains a basic arginine-rich patch (Figure 8B) that provides interaction determinants in non-canonical RNA binding proteins [75], as it is compatible with a putative role of PipX in RNA binding. It is therefore tempting to speculate that PipX may also be involved in the regulation of gene expression at the level of translation.

### 4.2. The PipX Synteny Network

**Figure 9:**
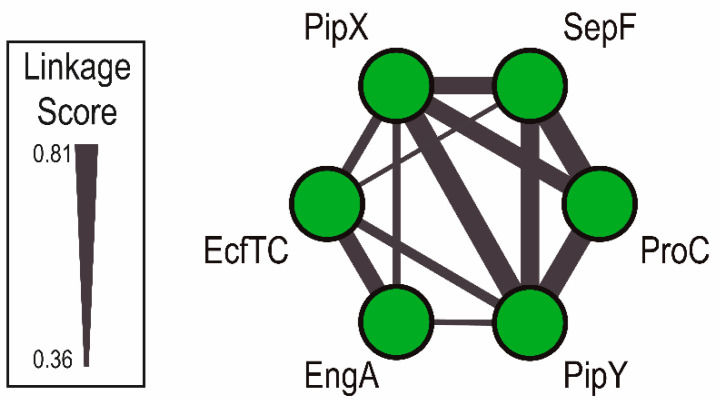
The PipX synteny network. Proteins are represented as nodes/circles, and syntenic relationships as lines whose width is proportional to the corresponding linkage score between nodes (adapted from [32]).

As previously mentioned, the discovery of PipX was due to the implementation of the “guilty by association” principle implicit in protein–protein interaction screenings. The same principle, applied in the context of gene linkage, led us to connect PipY with the cyanobacterial nitrogen regulation network. Given that in this phylum, most signaling proteins are encoded in monocistronic units, we have recently taken this “guilty by association” principle a step further, to look for genes that, independent of their operon structures, are closely associated with PipX or PipY in cyanobacterial genomes, and may thus be functionally connected. For this we turned to Cyanobacterial Linked Genome [32], a database generated on the basis of conservation of gene neighborhoods across cyanobacterial species, accessed through an interactive platform. The default outcome places PipX and PipY as part of a relatively robust six-node network (Figure 9), raising questions on the possible functional connections amongst them. Because one of the network nodes is EngA (YphC/Der/YfgK), a GTPase involved in ribosome assembly [76,77] that could play a role in the coordination of photosynthesis activity with ribosome function [78,79], current work is now focused in investigating its functional connections with PipX.

## 5. Conclusions

Cyanobacteria have developed sophisticated mechanisms to adapt their metabolic processes to important environmental challenges, like those imposed by the succession of days and nights. However, the regulatory mechanisms and molecular details behind the versatility and environmental adaptations of cyanobacteria are largely unknown, and the study of unique proteins like PipX, which is restricted to this phylum, provides an opportunity to unravel some of them. Particularly challenging is the identification of the biological processes and environmental contexts in which a rather promiscuous protein like PipX participates, a task that has been fueled by “guilty by association” approaches, in particular protein–protein interactions and genetic linkage. In this context, bioinformatic tools like the recently developed Cyanobacterial Linked Genome should also help to provide working hypotheses in the context of other proteins that may be unique to cyanobacteria.
